# *Pseudomonas aeruginosa* infection of avian origin: Zoonosis and one health implications

**DOI:** 10.14202/vetworld.2021.2155-2159

**Published:** 2021-08-21

**Authors:** Wafaa A. Abd El-Ghany

**Affiliations:** Department of Poultry Diseases, Faculty of Veterinary Medicine, Cairo University, Egypt

**Keywords:** antibiotic resistance, human, poultry, *Pseudomonas aeruginosa*, zoonosis

## Abstract

Zoonotic diseases are diseases that are transmitted from animals to humans and vice versa. *Pseudomonas aeruginosa* (*P. aeruginosa*) is a pathogen with zoonotic nature. Commercial poultry could be infected with *P. aeruginosa*, especially at young ages with great losses. Infection of embryos with *P. aeruginosa* induced death in the shell, while infection of chicks led to septicemia, respiratory and enteric infections, and high mortality. Humans are also highly susceptible to *P. aeruginosa* infection, and the disease is associated with severe lung damage, especially in immunocompromised patients. Chicken carcass and related poultry retail products play an important role in the transmission of *P. aeruginosa* to humans, especially after processing in abattoirs. Treatment of *P. aeruginosa* infection is extremely difficult due to continuous development of antibiotic resistance. The transfer of antibiotic-resistant genes from poultry products to humans creates an additional public health problem. Accordingly, this study focused on avian pseudomonad, especially *P. aeruginosa*, with respect to infection of poultry, transmission to humans, and treatment and antibiotic resistance.

## Introduction

The World Health Organization [1] defined any infection that is transmitted naturally from animals to humans or from humans to animals as zoonosis. Approximately 2.4 billion diseased cases and 2.7 million deaths in humans annually, along with the negative impact on livestock production, are related to these zoonotic infections, especially in low-income countries [2]. Most infectious and fatal diseases that infect humans are of animal or animal product origin [3]. There is a wide variety of bacterial zoonosis in poultry; among them, *Pseudomonas aeruginosa* (*P. aeruginosa*) infection has a direct public health hazard.

The significance of this study focuses on the problem of avian *P. aeruginosa* infection and its association with human health. *P. aeruginosa* is a ubiquitous organism that often presents in soil, water, and humid environments [4]. The bacterium can colonize soil and infect aquatic habitats, animals, and plants [5]. Moreover, *P. aeruginosa* is regarded as a normal inhabitant and opportunistic organism of avian spp. under normal environmental conditions. However, under stressors, such as immunosuppression, this organism becomes pathogenic and induces a clinical picture. Infection with *P. aeruginosa* in birds is associated with septicemia, respiratory signs, diarrhea, and deaths [6], with serious economic losses in the poultry industry [7]. The pathogen has been associated with high mortality in young chickens and late death in the shell of embryos [8].

In processing poultry plants, *Pseudomonas* spp. have been the predominant organisms on chicken carcasses [9,10]. Retail chicken products are incriminated as a primary source of *Pseudomonas* spp. for humans. Infection with *P. aeruginosa* induces severe pulmonary infection [11] and cystic lung fibrosis [12], especially in immunosuppressed persons.

In many countries, some antibiotics that are used in animal production could serve as essential medications for humans [13,14]. Due to the misuse of antibiotics in the animal-food production system, the global surge and spread of antibiotic resistance have been developed [15,16]. The food chain has been found to be an important reservoir of antibiotic-resistant *Pseudomonas* spp. [17]. Unfortunately, the rate of antibiotic resistance of *Pseudomonas* spp. in the food chain has been increasing worldwide [18].

Accordingly, this review article aimed to focus on avian pseudomonad, especially *P. aeruginosa*, with respect to infection of poultry, transmission to humans, and treatment and antibiotic resistance.

## Pathogen

Among pseudomonad, *P. aeruginosa* is the most common spp. causing poultry and human infections. Isolation and identification of *P. aeruginosa* revealed that the bacterium is Gram-negative, motile, non-spore former, and non-capsulated aerobic bacillus [19]. Isolates of *P. aeruginosa* can grow under aerobic conditions, producing β-zone of hemolysis on blood agar and non-lactose fermenter colonies on MacConkey agar. On the selective *Pseudomonas* agar base, the organism produces greenish pigment that is characterized by fruity smell. Serological examination of *P. aeruginosa* revealed that the most predominant serotypes were A, B, D, F, H, K, L, and M [20]. False-negative culture results due to overgrowth by other bacteria or presence of non-cultivable or mutant organisms induce some difficulties in using usual old techniques for detection of *P. aeruginosa* [21]. Qin *et al*. [22] recorded that the identification of *P. aeruginosa* with conventional methods takes a long time to perform and requires extensive hands-on work by technicians. Therefore, recent molecular methods have been developed for an accurate, specific, sensitive, and rapid diagnosis of the pathogen [23,24]. Molecular techniques, such as polymerase chain reaction (PCR), are used for the detection of *P. aeruginosa* DNA. In the study of Shahat *et al*. [25], the use of PCR with 16Sr DNA primers at 956 bp confirmed the existence of *P. aeruginosa* DNA in seven isolates collected from dead-in-shell embryos and broiler chickens in Egyptian poultry farms.

Several studies revealed the ability of *P. aeruginosa* strains to induce high mortalities, septicemic picture, un-absorbed yolk sac, pneumonia, necrosis of different organs, and enteritis in inoculated chicks [24,26,27]. Different virulent factors as *toxA, psIA*, and *fliC* genes can induce toxicity and pathogenicity of *P. aeruginosa* [24,28,29]. These factors inhibit protein biosynthesis, form biofilms, and have essential roles in the organism’s colonization and penetration of cells along with induction of necrosis and death of tissues [30-32]. The mechanism of *P. aeruginosa* infection has been studied by Li *et al*. [33]. After adherence and colonization of *P. aeruginosa* to the epithelium of the respiratory tract, macrophages become active, secrete different inflammatory factors, and activate the NF-κB pathway.

### Poultry infection

Birds of all ages are susceptible to *P. aeruginosa* infection, but young birds are severely affected. Birds can be infected with *P. aeruginosa* by a mechanical route through skin injury or use of contaminated needles during the vaccination process. Several factors, including the bird’s immune status and presence of other concomitant infections, may enhance the susceptibility to *P. aeruginosa* infection [6]. Infection with virulent strains of *P. aeruginosa*, especially in newly hatched chicks, resulted in dyspnea, diarrhea, dehydration, septicemia, and high mortality [24,34].

Different surveillance studies have been conducted to detect the incidence of *P. aeruginosa* infection among different chicken flocks. For instance, Satish and Priti [35] isolated *P. aeruginosa* at a rate of 12% from healthy chicks and 30% from diseased ones. Furthermore, *P. aeruginosa* was recovered in an incidence rate of 21.6% [36], 8.7% [26], and 17.6% [26] from broiler chicken flocks in different Egyptian governorates. Conversely, Hassan [37] isolated *P. aeruginosa* from diseased and dead broiler and day-old chicks in incidences of 25.3 and 10%, respectively. Moreover, *P. aeruginosa* has been detected in 42 of 480 (8.75%) broiler chicken samples [38] and 26 of 50 (52%) of dead-in-shell embryos [39]. Recently, 32 of 46 broiler chicken farms (69.57%) and 183 of 460 chicks (39.78%) were positive for *P. aeruginosa* [40]. Eraky *et al*. [24] recovered *P. aeruginosa* from 16 (8%) of 200 hatcheries and 17 (8.25%) of 206 chicken embryos samples.

### Human infection

*P. aeruginosa* is regarded as one of the most important pathogens that cause human opportunistic infections [41]. Besides, *P. aeruginosa* is considered a relevant and most frequently found pathogen causing severe acute nosocomial infections, especially in immunocompromised persons or patients in the intensive care unit [42]. After immunosuppression, the pathogen can cause many secondary infections, including chronic pulmonary lesions [11], cystic fibrosis of the lung, and other chronic underlying diseases [43].

Many studies indicated infection of humans with *Pseudomonas* spp. through occupational contact with poultry carcasses or related products. *Pseudomonas* spp. are regarded as important spoilage organisms that are present in spoiled poultry meat sold in retail settings [44,45]. Poultry processing plants are considered a potential source of human infection with *Pseudomonas*. After processing chicken carcasses, some *Pseudomonas* spp. in processed products were found viable and multiplied under cold air and water, as well as aerobic storage [44]. It has been documented that *Pseudomonas* spp. can spoil chicken’s carcass meat through production of proteolytic, saccharolytic, lipolytic, and biosurfactant changes, especially at the end of shelf life [45]. The predominance and persistence of *Pseudomonas* spp. in foods and on food processing surfaces may be related to the ability of these microorganisms to form a biofilm, which enhances their tolerance to harsh adverse conditions, including antimicrobial treatments [46,47].

### Treatment and antibiotic resistance

The sensitivity of *P. aeruginosa* strains to different antibiotic classes is extremely variable. For example, *P. aeruginosa* showed sensitivity to ciprofloxacin and gentamycin [39]. Moreover, sensitivity testing of *P. aeruginosa* strains of chicken origin showed susceptibility to levofloxacin, enrofloxacin, and danofloxacin in percentages of 81.25%, 59.375%, and 46.875%, respectively, while they showed complete resistance to nalidixic acid (100%) [40]. Egyptian chicken strains of *P. aeruginosa* showed 100% susceptibility to ciprofloxacin and norfloxacin, but 100% resistance to sulfamethazine, erythromycin, ampicillin, tetracycline, amoxicillin, and erythromycin [25]. Besides, Eraky *et al*. [24] demonstrated that all isolated *P. aeruginosa* strains (100%) were sensitive to ciprofloxacin, levofloxacin, and gentamycin but resistant (100%) to amoxicillin/clavulanic acid, doxycycline, and erythromycin.

Recently, the role of poultry products as a source of human foodborne pathogens, along with the transmission of antibiotic-resistant genes to humans, has emerged [16,48]. The presence of active pharmaceutical compounds from antibiotic manufacturing plants and serious pollutants of water and environment participated in the selection of antibiotic-resistant pathogens that pose a significant threat to the public [49].

Multidrug resistance to different antibiotic classes among *P. aeruginosa* isolates has been defined [50,51]. For instance, strains of *P. aeruginosa* that were isolated from diseased and dead chickens in Egypt showed multiple resistance to ampicillin, lincomycin, nalidixic acid, trimethoprim-sulfamethoxazole, chloramphenicol, tetracycline, and doxycycline [38]. Recently, the antibiotic resistance profile of *P. aeruginosa* isolates in poultry abattoirs in Nigeria showed a high level of resistance to carbapenems and cephalosporins [52]. These antibiotics have been employed in poultry production and human treatment [14].

High resistance of *P. aeruginosa* to most antibiotics may be relevant to the low permeability of the bacterial outer membrane lipoprotein (*oprL* gene) that is implicated in multidrug efflux transport system pump of RND-MFD-OMF encoded in its genome [53]. Furthermore, *P. aeruginosa* has various virulence factors, such as lipopolysaccharide, elastase, alkaline proteases, pyocyanin, pyoverdine, hemolysins, phospholipase C, and rhamnolipids. These factors are coordinated by a global regulatory system, which is activated by autoinducers involving *lasI* gene [54]. Moreover, *exoS, exoT, exoU*, and *exoY* genes of *P. aeruginosa*, which are important for production of toxic effector proteins into the cytosol of host cells, have been demonstrated.

Detection of some efflux pumps system genes, such as *oprJ, mexR, mexA, nfxB, ampC*, and *oprM*, in avian *P. aeruginosa* isolates was conducted, and the results revealed the presence of these genes with percentages of 100%, 71.4%, 85.7%, 76.2%, 45.2%, and 50%, respectively [38]. Furthermore, the presence of quorum sensing genes in *P. aeruginosa* regulates and provides information about the expression of virulence factors and pathogenicity of the organism [55]. *P. aeruginosa* quorum sensing genes, such as *LasI, LasR, Rh11*, and *RhIR*, and other virulence genes that inhibit protein biosynthesis, such as *toxA*, were detected in at prevalences of 85.7%, 92.9%, 66.7%, 80.9%, and 80.9%, respectively [38]. However, other studies detected that the incidence rate of *P. aeruginosa* virulence genes (*toxA, exoS, lasB*, and *lasI*) was 71.42% for each of them, while it was 100% for *oprL* gene [25].

Moreover, *P. aeruginosa* can acquire resistance by mutation through chromosome-encoded genes or horizontal gene transfers of antibiotic resistance determinants [18,56]. It is well known that *P. aeruginosa* harbors antibiotic-resistant plasmids, integrons, and transposons that transfer them to other spp. Rasamiravaka *et al*. [31] suggested that the opportunistic nature of *P. aeruginosa*, formation of biofilms, and presence of chronic infections may be the causes of multiple drug resistance. Therefore, intrinsic and acquired antibiotic resistance mechanisms cause difficulties in the treatment of *P. aeruginosa* infection [57]. Furthermore, antibiotic-resistant *Pseudomonas* spp. have been detected in chicken meat [58]. A recent study by Heir *et al*. [17] proved the occurrence of antibiotic resistance in psychrotrophic *Pseudomonas* spp. collected from retail chicken in Norway in a 26-year period. Moreover, *P. aeruginosa*-contaminated chicken carcasses in the abattoir harbor some resistance genes, such as metallo-b-lactamase (*bla*_IMP-1_, *bla*_IMP-2_, *bla*_VIM-1_, and *bla*_VIM-2_) and AmpC (*bla*_FOX_, *bla*_DHA_, *bla*_CMY_, and *bla*_ACC_), which are potential sources for the wide dissemination of antibiotic resistance [52].

Accordingly, both abattoirs and poultry farms are regarded as good grounds for the evolution and spread of antibiotic-resistant *P. aeruginosa* in the non-hospital environment [59]. Moreover, the litter of poultry that contains even a trace number of *P. aeruginosa* organism with antibiotic residues plays an important role in the spread of infection in the environments [38]. The presence of a high resistance pattern by *P. aeruginosa* isolates in poultry meat may be attributed to the continuous use of these antibiotics during the rearing period of birds.

The presence of resistance genes to quaternary ammonium compound disinfectants has also been detected. In *P. aeruginosa* strains from Egyptian chicks’ embryos and broiler chickens, the resistance rate to these disinfectants was 14.28% for *qacAB* and *qacCD* genes, while it was 100% for *qacED1* gene [25]. It was detected that genes coding resistance to disinfectants were combined decisively with genes coding for resistance to sulfonamides, trimethoprim, chloramphenicol, aminoglycosides, and β-lactams [60].

## Conclusion

From the abovementioned, it could be concluded that infection with *Pseudomonas* spp., especially *P. aeruginosa*, is a disease of significant importance for poultry production and human health. The pathogen is of zoonotic nature, which can be transmitted to humans after handling of poultry carcasses and related products in processing plants and abattoirs. The development of resistance to different antibiotics is common among *P. aeruginosa* strains, in either poultry or humans. Accordingly, strict supervision and application of laws to control antibiotic use in the food chain within the established safe levels should be conducted.

## Authors’ Contributions

WAA collected relevant literature, wrote the original draft, reviewed the manuscript, and approved the final version.
